# Examining tailoring as an implementation strategy for reducing healthcare-associated infections across European acute care hospitals (REVERSE): study protocol for a hybrid type 2 effectiveness-implementation trial

**DOI:** 10.1186/s13063-025-09132-x

**Published:** 2025-10-16

**Authors:** Bianca Albers, Laura Caci, Kathrin Blum, Greet Boland, Elena Carrara, Pilar Retamar Gentil, Aude Nguyen, Jack Pollard, Vered Schechner, Ashlesha Sonpar, Takuya Yanagida, Walter Zingg, Lauren Clack

**Affiliations:** 1 Institute for Implementation Science in Health Care, University of Zurich, Universitaetstrasse 84, 8006 Zurich, Switzerland; 2 Division of Infectious Diseases and Hospital Epidemiology, University of Zurich and University Hospital Zurich, Zurich, Switzerland; 3 Department of Developmental and Educational Psychology, University of Vienna, Vienna, Austria; 4 Division of Infectious Diseases, Geneva University Hospitals, Geneva, Switzerland; 5 National Institute for Antibiotic Resistance and Infection Control, Ministry of Health, Tel Aviv, Israel; 6 Faculty of Medical and Health Sciences, Tel Aviv University, Tel Aviv, Israel; 7 Department of Medical Microbiology, University Medical Center Utrecht, Utrecht, Netherlands; 8 Infectious Disease Section, Department of Diagnostics and Public Health, University of Verona, Verona, Italy; 9 Clinical Unit of Infectious Diseases and Microbiology, Virgen Macarena University Hospital, Department of Medicine, Instituto de Biomedicina de Sevilla (IBiS)/CSIC/, University of Seville, Seville, Spain; 10 AMR Modelling and Evaluations Team, AMR & HCAI, UK Health Security Agency, London, UK; 11 CIBER de Enfermedades Infecciosas, Instituto de Salud Carlos III (CIBERINFEC, ISCIII), Madrid, Spain

**Keywords:** Antimicrobial stewardship, Carbapenems, Nosocomial infections, Cross infection, Implementation science, Randomized controlled trial, Secondary care centers, Europe

## Abstract

**Background:**

Infection prevention and control (IPC) and antibiotic stewardship (ABS) represent promising approaches for reducing the prevalence of healthcare-associated infections (HAI) and antimicrobial resistance (AMR) in different healthcare settings. However, the combined use of IPC and ABS measures and ways to optimize their integrated implementation have been insufficiently considered and assessed. The REVERSE trial, funded by the European Union’s Horizon 2020 program, involves 24 acute care hospitals from four European countries, all with high rates of AMR and HAI. REVERSE aims to investigate whether the sequential implementation of an IPC and an ABS practice bundle is feasible and sustainable and whether externally guided tailoring as an enhanced implementation strategy leads to superior clinical and implementation outcomes compared to a basic implementation condition.

**Methods:**

REVERSE will be designed as a stepped wedge cluster randomized, hybrid type 2 trial, including an embedded implementation trial. Four cohorts of six acute care hospitals will sequentially enter the trial over 38 months and work to implement first IPC, and, after 1 year, add the ABS practice bundle. Simultaneously, hospitals will be provided basic implementation training and instructed to tailor their implementation, with half of the hospitals being self-guided in their tailoring, whereas hospitals in the enhanced implementation condition will receive time-limited external facilitation in practicing tailoring. Qualitative data will be collected longitudinally to investigate contextual conditions for implementing IPC and ABS locally and how they contribute to tailoring results. IPC and ABS feasibility, fidelity, and sustainability will be assessed together with tailoring fidelity using repeated measures. Retrospective, in-depth, explanatory case studies will be conducted to interpret hospital outcomes.

**Discussion:**

REVERSE is an extensive and complex effectiveness-implementation trial aimed at investigating tailoring effectiveness. It will contribute to the still scarce evidence base for this adaptive approach to integrating research-supported interventions into routine healthcare settings. By identifying pathways toward strengthening the integration of IPC and ABS practices at European acute care hospitals, REVERSE also has the potential to inform much-needed concerted efforts to combat the growing challenge of antimicrobial resistance in the region.

**Trial registration:**

In November 2021, the REVERSE study was registered with the “International Standard Randomised Controlled Trial Number” (ISRCTN) register under nr.12956554.

**Supplementary Information:**

The online version contains supplementary material available at 10.1186/s13063-025-09132-x.

## Background

Antimicrobial resistance (AMR) is a global challenge and represents a public health burden, increasingly threatening the effective treatment of infections [1]. In hospitals across the globe, multidrug-resistant organisms (MDROs) result in high rates of healthcare-associated infections (HAIs) in patients and increase both morbidity and mortality [2, 3]

AMR is a global problem, and Europe is one of many endemic hotspots. In 2019, 674,000 deaths were associated with or attributed to AMR across the WHO European Region, with the highest mortality rates found in Eastern (19.9 deaths per 100,000 attributable; 74.0 associated) and Central Europe (16.6 deaths per 100,000 attributable; 68.0 associated) [3]. A 2022/2023-point prevalence survey on HAIs and the use of antimicrobials at European acute care hospitals showed that 6.4% of patients received at least one antimicrobial for treating an HAI that year. The annual prevalence of patients with at least one HAI was estimated at 8%, equaling 4.3 million patients. Furthermore, 32% of microorganisms in microbiologically documented HAIs presented acquired resistance to first-line antimicrobials [4]. HAIs result in prolonged hospital admissions (1.9 to 19.9 excess days) [5–7], leading to a substantial cost burden estimated to be in the range of €4200–€29,909 per episode [6, 7]. In particular, Southern and Eastern Europe have high rates of AMR [2], with Greece, Italy, Romania, and Spain having a generally higher national antimicrobial resistance (and consumption) rate when compared to the European mean [2, 8].

Several interventions to combat AMR have been described in the literature, with infection prevention and control (IPC) [9–12] and antibiotic stewardship (ABS) [13–16] representing promising approaches for reducing AMR prevalence in different healthcare settings. *IPC* aims to reduce microbial transmission to patients and healthcare workers and prevent HAIs through preventive practices. These include the use of explicit IPC programs and guidelines; IPC training and education; HAI surveillance and the monitoring of adherence to IPC practice standards or guideline use; adherence to minimum staffing, workload, and bed occupancy ratios; access to adequate protective materials and medical equipment; and an appropriate, clean environment in healthcare facilities [17–19]. *ABS* describes a range of strategies promoting the judicious use of antibiotics in healthcare settings to reduce AMR. Examples are the use of ABS committees, audit and feedback, and guidelines for restricting the prescription and use of antibiotics [13, 20, 21]

Two features characterize the current literature on IPC and ABS. *First*, there remains a tendency to use, examine, and assess IPC and ABS separately. Based on the expectation that their braiding may further increase their effectiveness in combatting AMR, calls are increasingly made for the combined and integrated use of both programs [22–25], among others, to mutually leverage the experience and expertise of healthcare professionals involved in implementing either of the practice bundles [23]. These calls build on the awareness that improving IPC and ABS requires individual, organizational, and system-level behavior change only achievable through coordinated interdisciplinary efforts [24, 25]. *Second*, implementation quality (i.e., how these measures are concretely put into practice) continues to be highlighted as a central element insufficiently considered and assessed when investigating and adopting these practice bundles [17, 26–30]. For example, the quality with which behavior change interventions targeting healthcare professionals involved in IPC and ABS implementation were designed and reported was highlighted as an area for improvement in two recent systematic reviews [26, 30], each covering one practice bundle. Hence, there is a need to build further and enhance the evidence on the combined use and implementation of IPC and ABS. This is the goal of the REVERSE study (pREVention and management tools for rEducing antibiotic Resistance in high prevalence SEttings).^1^

REVERSE is a Horizon 2020-funded European stepped wedge hybrid type 2 cluster randomized trial aimed at assessing whether the combined use of IPC and ABS practice bundles is effective in reducing carbapenem-resistant HAIs across 24 acute care hospitals in Greece, Italy, Romania, and Spain. Hybrid studies combine research questions about the effectiveness of clinical interventions with those about their implementation [31–33]. The emphasis put on each of these two types of questions can differ, leading to three classifications of hybrid studies: one focusing primarily on evaluating intervention effectiveness while also gathering implementation information (type 1), one concentrating on the assessment of implementation strategies while also collecting data on intervention effectiveness (type 3), and finally, one weighing both types of research questions equally by testing the effectiveness of both a clinical intervention and an implementation strategy (type 2). The strength of these types of studies is the simultaneous—rather than sequential—examination of interventions’ effectiveness and implementation, having the potential to speed up the translation of research findings for use in routine healthcare settings [31]. In the past decade, hybrid studies have become more widely adopted in health and human services; however, in the field of infection prevention and control, they remain a rarity, with only a few examples of study protocols having been published [34, 35].

This REVERSE study protocol adds to this still scarce knowledge base and details the steps to be taken in conducting REVERSE as a hybrid type 2 trial.

The clinical effectiveness study of REVERSE has been registered with the “International Standard Randomised Controlled Trial Number” (ISRCTN) register.^2^ Next to assessing the effectiveness of IPC and ABS practice bundles, a key aim of the project is to examine whether and how implementation influences intervention effectiveness. The success of complex interventions such as practice bundles depends on the quality of their implementation. By “implementation,” we mean the “intentional use of strategies to introduce or adapt evidence-based interventions within real-world settings” [36].

Implementation strategies are “methods or techniques used to enhance adoption, implementation, and sustainability of a clinical program or practice” [37]. Ideally, these strategies are developed based on knowledge about prospectively identified barriers and facilitators to implementing practice bundles such as IPC and ABS, thereby continuously adjusting implementation work to the conditions of the context in which this work occurs [38]. This process has been labeled “tailoring,” i.e., “a careful analysis of barriers for implementation, matching of strategies to those barriers, followed by application and evaluation” [39]. Tailoring is the implementation approach that will be examined as part of REVERSE.

The evidence on the effectiveness of tailored implementation remains scarce and ambiguous. In a systematic review of 32 randomized controlled healthcare studies [40], the effects of tailored implementation were shown to be variable and only small to moderate, leading the research team to call for more trials examining tailoring in greater depth. A similar conclusion was drawn from a recent HAI-focused systematic review of implementation strategies used to prevent surgical site infections in abdominal surgery, showing that tailoring was rarely applied in included studies [41]. This, despite the promise tailoring holds for reducing HAIs—as documented through, e.g., the PSYGIENE trial [42, 43]. In this trial, tailoring was the selection of behavioral change techniques for training and feedback sessions with healthcare providers based on assessing psychological determinants of hand hygiene compliance. This led to a significant decrease in MDRO infections in intensive care units and hematopoietic stem cell transplantation units at a hospital in Germany.

Another example is the REACH study, reporting that tailoring, i.e., the site-specific operationalization of an implementation framework, including the assessment of local baseline practices, contextual factors, and evidence-practice gaps for pre-defined components of a cleaning bundle, led to improved cleaning practice and performance in 11 Australian hospitals [44, 45]. Hence, there is a need to further the evidence base on tailoring, its impact on HAIs, and its cost-effectiveness. Against this background, the following research questions form the core of the REVERSE implementation evaluation:

1. Is facilitated, tailored implementation of IPC and ABS superior to basic implementation of IPC and ABS, i.e., does it lead to significantly better clinical and implementation outcomes when compared with basic implementation?
2. What are the anticipated and encountered contextual barriers and facilitators to implementing IPC and ABS in acute care hospitals with a high prevalence of antimicrobial resistance in different regions of Italy, Spain, Greece, and Romania?
3. To what degree do REVERSE stakeholders perceive IPC and ABS as (a) usable, (b) feasible, and (c) sustainable?
4. Can IPC, ABS, and tailoring be implemented with fidelity?

## Methods

### Study design

REVERSE has been designed as a stepped wedge cluster randomized hybrid type 2 trial that will run from 2021 until 2026 in 24 acute care hospitals from four European countries (Italy, Greece, Romania, and Spain). Embedded in REVERSE is an additional implementation trial in which 12 of the 24 participating hospitals are randomized to an enhanced implementation condition.

### Guiding framework

Three frameworks will guide the conduct of the REVERSE implementation trial, including the context-specific tailoring (CST) model [46], the Consolidated Framework for Implementation Research (CFIR) version 2.0 [47], and an implementation outcomes framework [48].

**Fig. 1:**
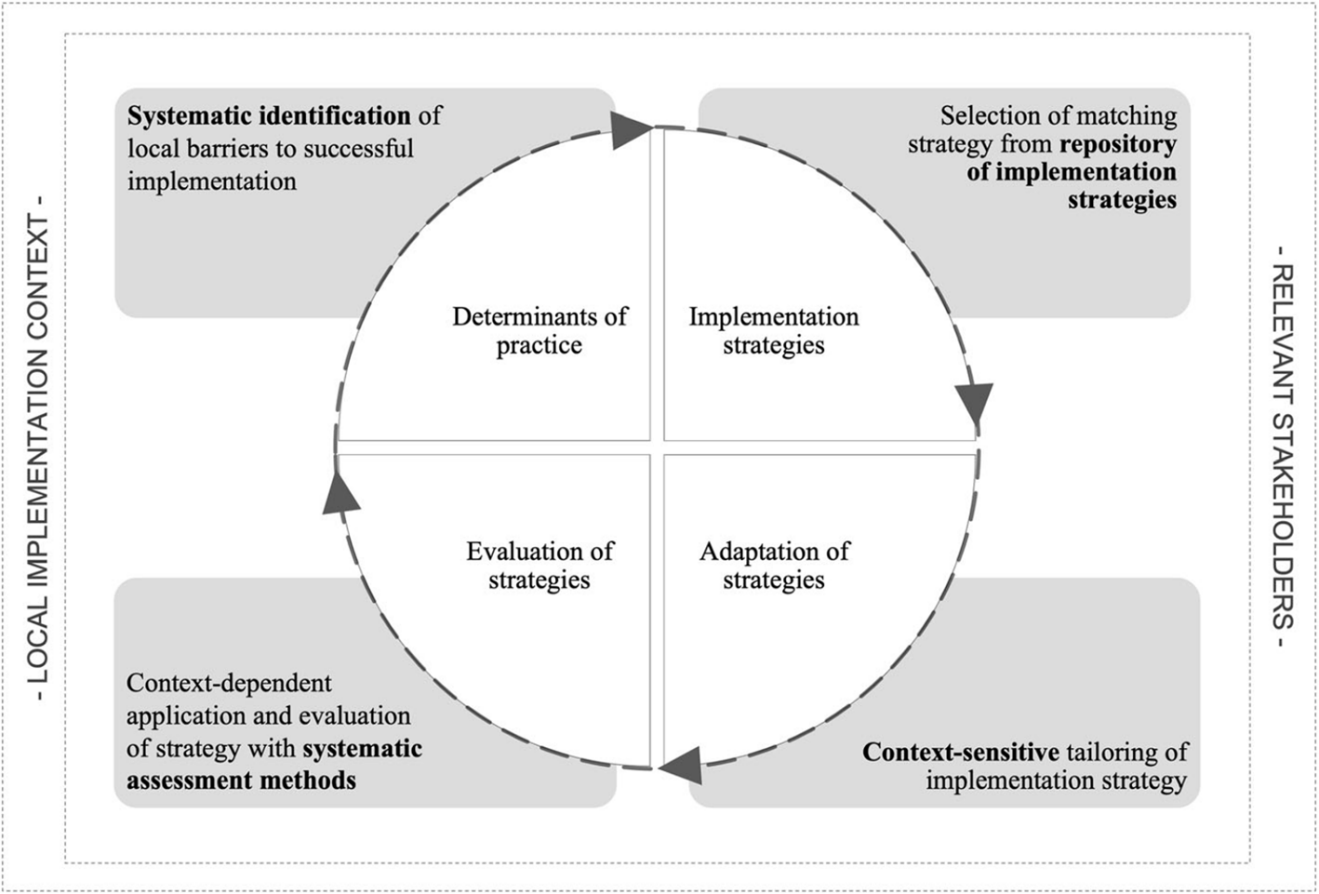
Process of context-specific tailoring as applied in the REVERSE project

To conceptualize the process of tailoring that forms the core of this trial, we will use the CST model shown in Fig. 1, which was initially developed for the *ImpleMentAll* project, a cross-European implementation study conducted in the field of mental health [46, 49].

This figure is unadapted from its original version and licensed under a Creative Commons Generic License (CC BY 4.0 OA). It is attributed to Bührmann et al. [46]

This figure describes tailoring as a prospective activity that involves (a) identifying locally relevant barriers to implementation, (b) selecting matching implementation strategies, and (c) operationalizing these strategies to fit local conditions, followed by (d) their context-dependent use and ongoing evaluation of this use. The CST will inform all research activities related to tailoring, including its operationalization, education, and evaluation.

The CFIR version 2.0 [47] will guide all research activities for examining and understanding barriers and facilitators to REVERSE practice bundle implementation. The CFIR was developed as a determinant framework aimed at helping to explain implementation outcomes. The updated version of the CFIR is structured into five domains of factors that can influence the implementation of innovations in healthcare, including *innovation*, *outer setting*, *inner setting*, *individuals*, and *implementation process*. Additional (sub)-constructs form each domain, further detailing the domain-specific factors affecting implementation work. *Structural characteristics*, *relational connections,* or *culture* are examples of constructs in the inner setting domain, whereas *critical incidents*, *policies*, *laws*, or *external pressure* represent a sample of outer setting constructs.

**Fig. 2:**
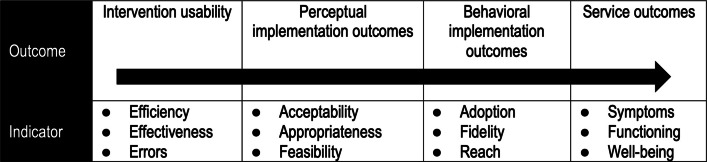
Association of intervention usability with implementation and service outcomes

Finally, the implementation outcomes framework to be used was developed based on established implementation outcomes [48] and arranges these in relation to each other [50], emphasizing that intervention usability precedes perceptual implementation outcomes, which again precede behavioral implementation outcomes as outlined in Fig. 2. This framework will guide the selection and analysis of implementation outcomes to be measured in this study.

This figure was adapted from its original version by Lyon & Bruns [50].

### Participating hospitals and their randomization

Based on findings and modeling of the 2016/2017 ECDC point prevalence survey [51], mean estimated incidence densities of HAIs due to a composite index incorporating carbapenem-resistant Enterobacterales (CRE), carbapenem-resistant Pseudomonas aeruginosa (CRPA), and carbapenem-resistant Acinetobacter baumannii (CRAB) combined for Greece, Italy, Romania, and Spain were 2.99/1000 patient-days, 0.73, 0.62, and 0.51, respectively. Considering the lowest incidence density of 0.5/1000 patient days, an intra-cluster correlation of 0.9, four randomization steps, and 25,000 admissions per year on average, it is estimated that 24 acute care hospitals will provide sufficient power (standard power [80%] and alpha [0.05] values were used) to perform all relevant comparisons for the primary outcome as specified in Table 4. This includes the hypothesis that enhanced implementation will have an added effect on the primary outcome on top of a 35% impact of the combined IPC- and ABS-bundles, leading to an additional 15% in HAI reduction.

The 24 REVERSE hospitals were recruited based on the aforementioned 2016/2017-point prevalence survey [51], reflecting a high prevalence of MDROs in these hospitals, specifically of CRE, CRPA, or CRAB. These carbapenem-resistant organisms (CROs) represent a particularly severe threat to the health of patients due to the limited availability of treatment options. Each hospital will participate in REVERSE with approximately 350 beds that are part of intensive care, internal medicine, hematology-oncology, or surgery (including transplant) units. Pediatric and obstetrics/gynecology wards are excluded.

**Fig. 3:**
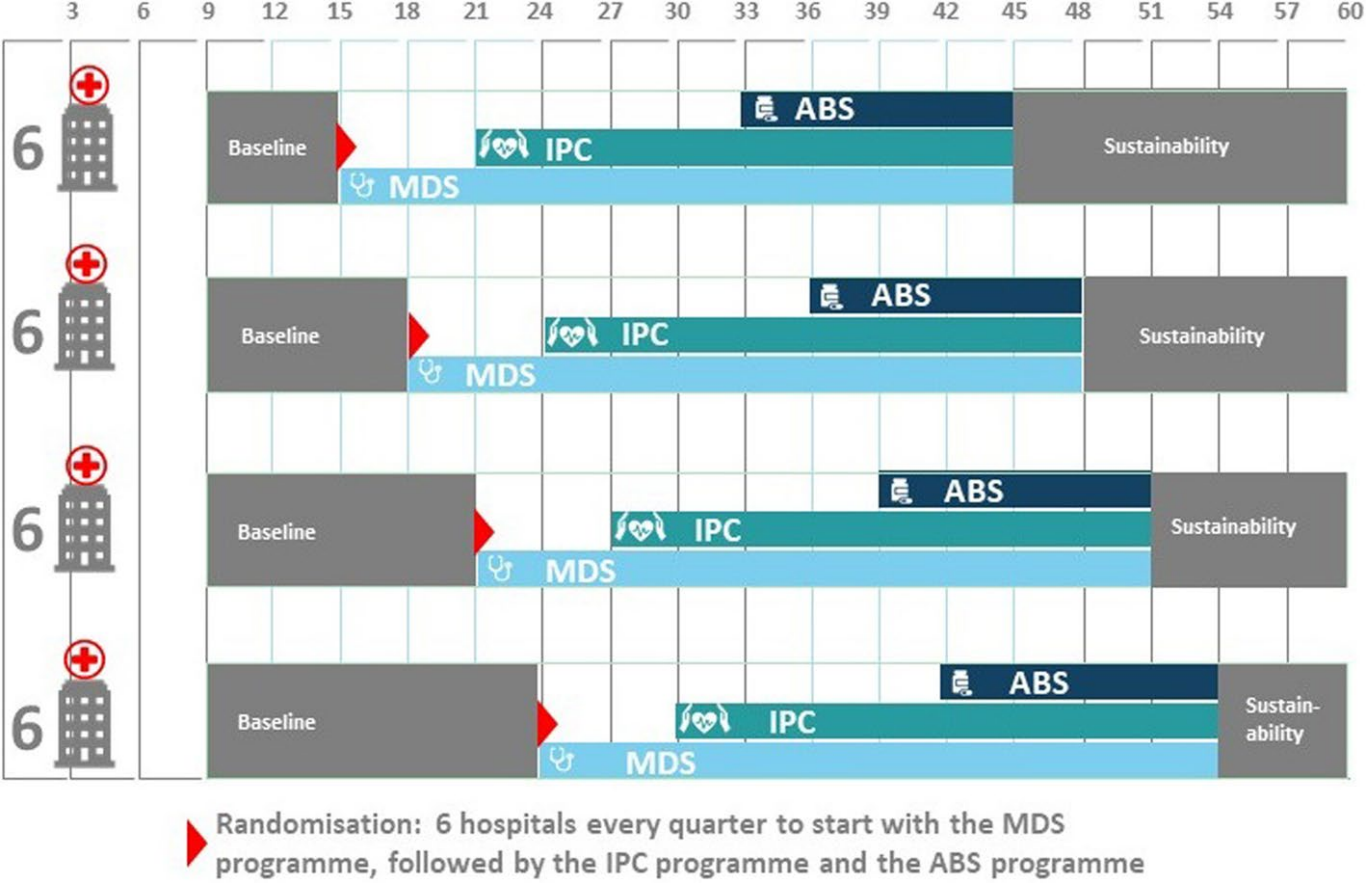
The REVERSE trial—randomization and cohorts

The trial coordinator (AS) will randomize hospitals to four cohorts of six hospitals, stratified by country (using the Stata *randtreat* package) and implementation condition (using the R *minirand* package). Each cohort will contain hospitals from every country and an equal number in both implementation arms. Hospitals will not be informed about which arm they belong to. The four cohorts will enter the trial at four different time points, as outlined in Fig. 3. Hospitals will first implement IPC and, 1 year later, begin the implementation of ABS.

### Clinical interventions

To clarify microbiological capacity, all hospitals will first undergo a Microbiology and Diagnostic Stewardship (MDS) intervention and complete two surveys: the first about laboratory workflows, particularly those for processing blood cultures, lower respiratory tract specimens, and screening rectal swabs. The second survey is an external audit that involves the workup of 14 standard strains to assess hospitals’ microbiology capacity regarding resistance and antimicrobial susceptibility testing. Where limitations are identified in this auditing, hospitals will receive recommendations for improving their microbiology capacity related to, e.g., the use of rapid diagnostics, opportunities for improving workflows, and similar.

Hereafter, hospitals will sequentially implement two bundles of clinical practices, one focused on infection prevention and control and one on antibiotic stewardship. The basic practices included in each bundle are listed in Table 1.

**Table 1:** REVERSE practice bundles and their components

#	Infection prevention and control bundle (IPC)	#	Antibiotic stewardship bundle (ABS)
1	SOP1: Hand hygiene (HH)—based on WHO standards “5 moments of hand hygiene”	1	Establishment of a multidisciplinary stewardship committee with regular meetings
2	SOP2: Contact precautions (CP)—use of personal protective equipment; separation of patients; limiting transport/movement of patients; allocation of patient care equipment; enhanced cleaning of patient rooms	2	Guidance document on syndrome-specific treatment pathways
3	SOP3: Isolation & cohorting (IC)—territorial separation of MDRO carriers; use of dedicated staff to exclusively manage isolated/cohorted patients	3	Dedicated recommendations for new drugs
4	SOP4: Active surveillance (AS)—patient screening for MDROs on admission and/or at regular intervals	4	Training on judicious antibiotic prescription
5	SOP5: Healthcare environmental hygiene (HEH)—development and use of protocols for monitoring and auditing cleaning practices	5	Audit and feedback on compliance to guidance on antibiotic use
6	SOP6: Outbreak management (OM)—development and use of OM protocol and team processes	6	Regular stewardship rounds in high-risk settings (intensive care, hematology-oncology, transplant units)
7		7	Pathways for integration of antibiotic consumption reporting to the stewardship policies

International, professional society standards, national or local guidelines and recommendations, and existing literature informed the selection of these practices for each bundle. For ABS, a particular focus was on non-restrictive interventions (e.g., guideline provision, audit and feedback, selection of ward champions) deemed more sustainable over time and less prone to unintended consequences such as delay in treatment or negative professional culture [16].

Each REVERSE hospital is expected to initiate the implementation of at least three IPC standard operating procedures (SOPs) within year 1 following the basic training in these SOPs. This implementation will likely begin at different baselines, with some hospitals already applying versions of some of the SOPs listed and others not yet using them.

To implement ABS practices, each REVERSE hospital will define specific antibiotic prescription targets in a medical (e.g., treatment of asymptomatic bacteriuria) and a surgical area (e.g., surgical prophylaxis). For these prioritized targets, hospitals will perform an audit and provide feedback on at least 20 antibiotic prescriptions per month in each area.

### Basic vs. enhanced implementation

The implementation strategy to be assessed in REVERSE is tailoring, with hospitals in the basic condition tailoring their IPC and ABS implementation independently, while those in the enhanced condition participate in time-limited external facilitation activities to inform their tailoring practice. The details of these two implementation conditions, of which hospitals are unaware, are outlined in Table 2 and will be applied sequentially, cohort by cohort. A more detailed strategy specification based on principles defined by Proctor et al. [37] is provided in Appendix A.

**Table 2:** Basic and enhanced implementation in REVERSE

Element	Basic implementation	Facilitated tailoring (enhanced)
(1) Basic cohort training • Held twice, in the month prior to commencing practice bundle implementation	- Participation in one 1.5-day practice bundle implementation workshop with three hospital staff participating in person and additional hospital collaborators participating online - Implementation topics covered: an introduction to implementation science; the role of the implementation team; engagement of hospital collaborators; goal setting in IPC/ABS implementation; identifying determinants; selecting strategies; monitoring implementation practice	
(2) Curated electronic collection of implementation resources • Shared in the week post (1)	Provision of a collection of electronic materials about practices bundles and their implementation; additional resources available on demand	
(3) Forming of a multidisciplinary implementation team	Hospitals in both conditions are encouraged to establish a local multidisciplinary implementation team for each practice bundle	
(4) REVERSE Kick-off calls • Held 2 to 3 weeks post (1)	One 60–90 min online meeting with hospitals in the basic condition focused on supporting local implementation commencement	One 60–90 min online meeting with hospitals in the enhanced condition focused on supporting local implementation commencement and preparing for implementation calls
(5) REVERSE implementation calls • Beginning 2 weeks post (3)	Held only when requested	Three online group calls with three participating hospitals • Call 1: 90 min; 3 weeks post (3); focus on goal setting and determinant identification • Call 2: 60 min; 6 weeks post (3); focus on determinants and strategy selection • Call 3: 60 min; 9 weeks post (3); focus on strategy operationalization and implementation monitoring Further support available on demand
(6) Regular implementation progress reporting	- Quarterly submissions of updated versions of the REVERSE Implementation Tool (RIT) summarizing recent implementation activities and results - Feedback on the RIT is only provided on demand	- Quarterly submissions of updated versions of the REVERSE Implementation Tool (RIT) summarizing recent implementation activities and results - As a standard, the implementation research team returns RIT with feedback, commentary, and advice within 2 to 3 weeks after submission
(7) Implementation check-in • One per hospital; held 6 months post commencing practice bundle implementation	One 60 min online meeting with the hospital’s implementation team with a focus on how practice bundle implementation is perceived, progresses, and can be strengthened	

#### Implementation and intervention training and materials (basic and enhanced)

Hospitals in both conditions will attend two introductory practice bundle and implementation training workshops, one before beginning the IPC implementation and one before initiating ABS implementation. A team of REVERSE researchers will deliver this training component, including at least two IPC or ABS specialists, two senior implementation experts, and two junior researchers with clinical and implementation experience. Workshops will be held in a hybrid format, with up to three staff members per hospital attending in person and additional hospital staff participating online. While hospitals will decide whom to send to the basic cohort training, the REVERSE research team will recommend the roles listed in Table 3 for in-person participation in the training and the subsequent implementation team formation.

**Table 3:** Recommendations for selecting trainees for in-person attendance in basic cohort training

IPC basic cohort training	ABS basic cohort training
One IPC specialist responsible for a hospital’s IPC activities	One ABS clinician involved in/leading hospital’s ABS program
One physician involved in IPC activities	One pharmacist
One other person involved in IPC and MDRO control	One microbiologist

All sites will have access to a *collection of electronic materials and resources* that introduce the role of implementation in IPC and ABS and provide guidance on how to implement practices actively and intentionally in hospital settings.

#### Formation of local implementation teams (basic and enhanced)

As part of the workshops, all hospitals will be encouraged to form a local implementation team [52] responsible for coordinating and facilitating all activities related to implementing both practice bundles. Guidance on key considerations in forming implementation teams will be provided during workshops.

#### Implementation kick-off calls (basic and enhanced)

Two to three weeks after these workshops, an *online kick-off meeting* with all sites in a cohort, separated by implementation condition, will be held to support hospitals with the first steps in practice bundle implementation. Hospitals’ local IPC and ABS implementation teams will attend these kick-off calls. These teams would be formed following the basic training and will likely vary in size and composition.

#### Facilitated tailoring (enhanced)

Additionally, implementation teams from hospitals in the enhanced implementation condition will receive *three 1-h online educational group sessions* focused on learning and discussing how to prospectively tailor local implementation work, with each meeting focusing on a particular aspect of tailoring. Implementation team members from all three hospitals in the enhanced implementation condition of a cohort will attend these sessions together to enable knowledge exchange across hospitals, each of which will be located in a different country and representing a different hospital culture. It will also be emphasized for these sites that further support from the research team can be requested at any time, be it as part of implementation-related brainstorming, problem-solving, data analysis, or similar activities. Implementation planning tasks will be assigned between meetings. Finally, the REVERSE research team will provide implementation teams from hospitals in the enhanced condition with feedback on their quarterly reporting of implementation progress for both practice bundles.

Groups of at least three research team members, involving minimum one clinical and two implementation experts, will deliver kick off and implementation calls.

#### Quarterly implementation reporting (basic and enhanced)

Throughout the work with implementing practice bundles, local implementation teams will be reporting their implementation work quarterly, using the REVERSE Implementation Tool (RIT) [53]. The RIT is a Microsoft Word-based template developed for the REVERSE study based on the CST (see Fig. 1). The RIT will guide hospital implementers through questions about the prioritized practices, the goals intended to be achieved, the determinants expected to influence implementation, and the strategies used to navigate and utilize these. Further details are provided in the measures section below.

While sites in the enhanced implementation condition will receive feedback on their RITs within 2–3 weeks after the submission deadline, combining input from members of the REVERSE implementation research team with that from the clinical IPC or ABS team, hospitals in the basic condition, unaware of this difference, will only receive this feedback on demand.

#### Six-month implementation check-in

Finally, an implementation check-in will be held with all sites 6 months post the commencement of the practice bundle implementation. These check-ins will be held online with a single hospital’s implementation team to learn about the status quo of implementation activities, explore whether support is needed, and discuss plans for progressing implementation.

### Study participants

Each hospital will form a practice-bundle-specific, local REVERSE implementation team, with likely participants including, for example, infectious diseases specialists, other physicians, infection prevention and control nurses, epidemiologists, pharmacists, or microbiologists. In addition, and supported through funding provided through the REVERSE trial, each REVERSE hospital will identify a REVERSE study nurse and a REVERSE study physician, who will be involved in local implementation activities. This team, in its entirety or in smaller groups of key team members, will be the REVERSE research team’s central interface with each hospital and hold the overarching responsibility for implementing IPC and ABS practice bundles across participating wards.

These responsibilities include recruiting additional study participants for inclusion in data collection by using purposive sampling throughout the trial. Study participants will be staff working on wards that the hospitals’ implementation teams decide to involve in local implementation efforts. Since hospitals will select different practice bundle SOPs and targets for implementation, these participants, including frontline and head nurses, nurse assistants, physicians, specialists, cleaners, and others, will vary between and within hospitals over time.

### Study outcomes, measures, and their administration

The REVERSE implementation evaluation focuses on different targets and outcomes. These are listed in Table 4, according to the research question they are linked to, together with the measure to be used for their assessment and the frequency with which they will be administered.

**Table 4:** REVERSE implementation evaluation outcomes, measures, and their administration

#	Research question	Outcome/target	Outcome measure	Administration
1	Is facilitated, tailored implementation of IPC and ABS superior to basic implementation of IPC and ABS, i.e., does it lead to significantly better clinical and implementation outcomes when compared with basic implementation?	Primary: Healthcare-acquired infections (HAIs)	- Incidence density per quarter (N/1000 patient-days) of HAIs due to CRE, CRPA, and CRAB, combined in a composite index - Incidence density per quarter (N/1000 patient-days) of HAIs due to ESBL, MRSA, and VRE combined in a composite index	- HAIs: prospective data collection for all microbiologically confirmed HAIs by the organisms of interest in all participating REVERSE wards
		IPC/ABS usability	- Intervention Usability Scale (IUS)	- Months 3, 6, 10, and 20 post IPC start, and months 3, 6, and 10 post ABS start
		IPC/ABS feasibility	- Feasibility of Interventions Measure (FIM)	- Months 3, 6, 10, and 20 post IPC start, and months 3, 6, and 10 post ABS start
		IPC/ABS sustainability	- Clinical Sustainability Assessment Tool (CSAT)	- Months 3, 6, 10, and 20 post IPC start, and months 3, 6, and 10 post ABS start
2	What are the anticipated and actual contextual barriers and facilitators of implementing IPC and ABS in acute care hospitals with a high prevalence of antimicrobial resistance located in different regions in Italy, Spain, Greece, and Romania?	Implementation context/determinants	- REVERSE Contextual Factors Survey (CFS) - CFS follow up interview with local IPC/ABS team	- 8 months prior to IPC and ABS start - 4 months into IPC and ABS implementation
			- Online check-ins with local IPC/ABS team	- 6 months into IPC and ABS implementation
			- Site visits including individual stakeholder interviews and hospital rounds	- Enhanced sites only - One site visit prior to IPC start - One site visit prior to ABS start
3	To what degree do REVERSE stakeholders perceive clinical interventions as (a) usable, (b) feasible, and (c) sustainable?	IPC/ABS usability	- IUS (per the above)	- Months 3, 6, 10, and 20 post IPC start, and months 3, 6, and 10 post ABS start
		IPC/ABS feasibility	- FIM (per the above)	- Months 3, 6, 10, and 20 post IPC start, and months 3, 6, and 10 post ABS start
		IPC/ABS sustainability	- CSAT (per the above)	- Months 3, 6, 10, and 20 post IPC start, and months 3, 6, and 10 post ABS start
4	Can IPC, ABS, and tailoring be implemented with fidelity?	IPC/ABS fidelity	- IPC/ABS process indicators	- Quarterly reporting by sites
		Tailoring fidelity	- REVERSE Implementation Tool (RIT) assessment	- Quarterly RIT updates from sites

The clinical effectiveness of implemented measures will be monitored by determining the prevalence of CRE, CRPA, and CRAB at three different time points. At each point, rectal swab samples (250 per hospital) will be sent to a central lab for resistance determination.

Based on the aforementioned implementation outcome framework [50], the research team agreed to prioritize the assessment of intervention usability in combination with a minimum of one perceptual and one behavioral implementation outcome. This led to the selection of feasibility and fidelity as key assessment targets for REVERSE clinical interventions, partly to understand to what degree REVERSE clinical interventions are perceived as easy to use across a multitude of contexts, partly to inform the interpretation of study results for which it was deemed important to understand whether interventions are practiced as intended. For the same reason, it was decided also to measure tailoring fidelity. Additionally, stakeholders’ perception of REVERSE interventions’ sustainability was selected as an implementation outcome to learn from the trial whether interventions will require further adaptation to be maintained and scaled up.

The measures listed in Table 4 represent a combination of standardized and tailor-made quantitative, qualitative, and mixed-method instruments and tools.

The *quantitative measures* used combine strong psychometric with pragmatic properties. They include the *Intervention Usability Scale* (IUS) [54], the *Feasibility of Intervention Measure* (FIM) [55], and the *Clinical Sustainability Assessment Tool* (CSAT) [56]. Together, these measures will be used to assess the extent to which healthcare professionals perceive the IPC and ABS practice bundles as being usable, easy to carry out, and sustainable in their local setting. A search was conducted, and measure developers were contacted to identify existing translations of these measures into the four REVERSE languages.

This led to the identification of existing translations, as summarized in Table 5. For the remaining languages, the REVERSE research team followed standard guidance for forward- and back translations [57] involving a broad range of native speakers to support the translation work.

**Table 5:** Quantitative measures and their translation for the REVERSE trial

Measure	Existing translation used	Languages for which a full for-/backward translation was conducted
IUS	- The IUS is based on the System Usability Scale (SUS), for which a Greek and an Italian version existed - The IUS differs from the SUS in the use of one word (the former uses “system,” the latter “intervention”). The existing translated versions of the SUS only required to replace “system” with “intervention” in the respective language	Romanian, Spanish
FIM	Greek	Italian, Romanian, Spanish
CSAT	Spanish	Greek, Italian, Romanian

For each language, the three measures will be integrated into one tool that will be administered with all REVERSE hospitals (*N* = 24) three times during IPC and three times during ABS implementation, namely in months 3, 6, and 10. Since the duration of IPC implementation is set for 2 years, one additional measurement will be conducted in month 20 to assess whether stakeholder perceptions change at a later stage of the implementation and a time when the work with ABS has been added. At each point of administration, the REVERSE study nurses will be asked to distribute a link to the survey among implementation team members and other relevant hospital staff involved in the local implementation of both practice bundles in their respective hospitals. The intended minimum sample size per round of survey administration is *n* = 240, i.e., ten respondents per hospital and round of administration. Since hospitals’ implementation work will change over time, it is not the intention to include the same respondents in every round of survey administration.

*Intervention fidelity* describes “the degree to which interventions are put into practice as intended” [58], which in the REVERSE trial refers to both practice bundles. To measure practice bundle fidelity—within the field of IPC and ABS, often referred to as *adherence* [38, 59]—a set of clinical process indicators has been defined for each practice bundle, thereby following common approaches to monitoring the quality of care in this field [60]. Table 6 provides an overview of these clinical process indicators for REVERSE IPC.

**Table 6:** Clinical process indicators for REVERSE IPC

SOP*	**Process indicator**	**Reporting**
**HH**	WHO Hand hygiene Self-Assessment	Annually
	Alcohol hand rub consumption	Quarterly
	HH compliance	Quarterly (Minimal requirement: 3 HH observations sessions per unit quarterly)
**CP**	% of patients requiring CP who are actually under CP	Quarterly (Minimal requirement: 1 observation per ward per quarter)
	% compliance (infrastructure, placement, equipment)	Quarterly (Minimal requirement: 10 observations [i.e., 10 patients] per quarter)
**IC**	% of patients requiring IC who are actually under IC (according to hospital’s own policy of who requires IC). Specified by MDROs	Quarterly (Minimal requirement: 6 point-prevalence-audits per quarter)
	Speed of isolating or cohorting for newly identified Multi-Drug-Resistant-Gram-Negative-Bacteria (MDR-GNB) carriers	Quarterly (Minimal requirement: Point prevalence audit of 10 patients isolated or in a cohort per quarter)
	# of failures of containment within a cohort, based on clinical and screening samples	Quarterly
**AS**	Number of patients screened upon 1000 admissions/year, specified by CRE/CRPA/CRAB	Annually (Minimal requirement: Each hospital should upgrade screening activities according to local epidemiology and problem areas, e.g., increasing targeted screening in high-risk areas)
	MDRO positivity percentage/year, specified by CRE/CRPA/CRAB	Annually
**HEH**	None	(Minimal requirement: 2 audits per cleaner per year; all REVERSE units)
**OM**	None	As needed

^*^HH Hand Hygiene, *CP* Contact Precautions, *IC* Isolation and Cohorting, *AS* Active Surveillance, *HEH* Healthcare Environmental Hygiene, *OM* Outbreak Management

To monitor fidelity to ABS practices, hospitals will be required to report their audit and feedback activities using a pre-defined electronic case report form (eCRF) hosted on Research Electronic Data Capture (RedCap), a secure, web-based software platform designed to support data capture for research studies. The eCRF will enable the research team to track to what degree audits and feedback were conducted twice monthly and on a minimum number of twenty prescriptions. Clinical process indicators for hospitals’ ABS implementation teams to use are listed in Table 7.

**Table 7:** ABS process indicators for use in audit and feedback

Quality indicator	Definition
Guideline adherence—empiric treatment	The prescription of ABs adheres to guideline recommendations for
	• Antibiotic choice
	• Dosage
	• Frequency
	• Route
	• Duration
Guideline adherence—surgical prophylaxis	The prescription of antibiotics (AB) adheres to guideline recommendations for
	• Antibiotic choice
	• Timing of AB administration pre surgery
	• Dose
	• Repeat AB administration during operation
	• Overall duration
De-escalation	De-escalation of AB use is performed as early as possible and at least within 24 h post definitive culture results and antibiogram availability
Switch to oral	A switch to oral AB therapy is considered after the initial 48–72 h of intravenous treatment in patients with a good clinical response and adequate intestinal absorption
Duration	The overall duration of AB therapy is in line with guidelines and discontinued if there is no clinical evidence of infection. A review of AB therapy is conducted after the first 72 h or as soon as microbiological results are available
Appropriateness of culture	Appropriate microbiological samples—based on the possible site of infection—have been collected before initiating antibiotic therapy
Dose and intervals	The antibiotic dose and administration frequency adheres to guidelines
Antibiotic plan	A plan for the AB prescription is included in the medical documentation. It includes
	• The rationale for initiating antibiotic therapy
	• The rationale for guideline deviation
	• Name of the AB
	• Dosage
	• Administration interval
	• Treatment duration
	• Administration route
Early stop or no start	In the absence of clinical evidence of infection, AB therapy is discontinued

Sites will be requested to report quarterly clinical process indicator data for both practice bundles for the practices they have prioritized for implementation. Based on this data, site-based IPC/ABS fidelity profiles will be developed for each hospital.

To measure sites’ *implementation fidelity*, i.e., the degree to which hospitals’ implementation teams used tailoring as intended, the REVERSE Implementation Tool (RIT) will be used. As described elsewhere [53], the RIT was developed based on the CST [46] as an implementation reflection and planning tool for REVERSE sites and a means for ongoing reporting of implementation considerations and activities (i.e., implementation log). It is structured into questions for implementation teams to respond to in prioritizing their SOPs and planning and monitoring their implementation work. Next to asking about the SOPs or targets in focus, the RIT also includes guiding questions about hospitals’ implementation team composition, intended stakeholders for engagement, identified implementation determinants, planned implementation strategies, and the intended plan for monitoring local implementation. Hospitals will be asked to document their implementation practice using the RIT throughout the REVERSE study for their work with IPC and ABS once per quarter. All versions of a hospital’s IPC and ABS RITs will be collected by the research team for analysis, with an intended N of nine IPC RITs and five ABS RITs per hospital, leading to a maximum of 336 RITs to be analyzed for evidence that sites (or local implementation teams) have engaged in tailoring implementation activities to their local context.

The *REVERSE Contextual Factors Survey (CFS)* is a 46-item mixed-method questionnaire developed to explore the local context within which REVERSE practice bundles will be implemented. The CFS exists in an IPC and an ABS version, each of which has been informed by the CFIR, with all framework domains being represented by multiple items. The CFS also includes questions related to implementation as usual, i.e., the practices used to implement IPC and ABS measures before REVERSE.

The CFS is divided into blocks of statements, which respondents rate on a 5-point Likert scale from 1 (*strongly disagree*) to 5 (*strongly agree*) or comment with *yes*/*no*. For some statements, free text fields provide opportunities to explain or detail responses.

Following an internal review of the original English version of the survey among REVERSE research team members, three cognitive interviews with external volunteers were conducted. For the IPC version, interviewees included an IPC physician, an IPC nurse, and an Infectious Diseases specialist to identify language and content problems in the survey. This led to 27 modifications of the CFS. For the ABS version, cognitive interviews were conducted with four external volunteers, including three Infectious Diseases physicians and one Antimicrobial Stewardship pharmacist, leading to 35 changes made to the ABS CFS. The resulting versions were then translated into the four REVERSE languages using the services of professional translation agencies.

Both versions of the CFS will be administered with all REVERSE hospitals (*N* = 24) 8 months *before* and 4 months *into* IPC and ABS implementation, involving a minimum of three respondents with central responsibilities for local IPC and ABS work. These respondents will be identified using the purposive sampling approach described above (*n* = 72 per round).

Following the receipt of survey results, *CFS follow-up interviews* will be held with central hospital IPC and ABS contact persons from all REVERSE sites. These interviews aim to explore survey results further and deepen the research team’s understanding of hospital contexts. Interviews will be held in English and online, using the videoconferencing platform Zoom,^3^ and will involve two REVERSE research team members. One member will lead the interview, and the other will take thorough notes of interviewees’ responses.

Two rounds of *country-based, in-person site visits* will be conducted by the REVERSE research team only involving hospitals in the enhanced condition (*n* = 12). The visiting team will include a minimum of one clinical IPC or ABS specialist, one senior implementation expert, and two junior researchers with clinical and implementation experience. One visit will precede the commencement of IPC and one that of ABS implementation. The purpose of these visits is to gain further contextual insights that—together with CFS results—can inform the research team’s understanding of local conditions for implementing the REVERSE practice bundles and the support provided to sites over time. Site visits and their preparation will be based on the site visit standards developed initially by Patton [61] and further refined by Kenney et al. [62]. They will primarily include individual interviews with local healthcare professionals involved in IPC and ABS work. Observational activities will focus on exploring the physical, structural, and processual conditions for IPC and ABS implementation at each hospital and not involve patient-focused activities. Where applicable, relevant artifacts (e.g., local guidelines, posters) will also be collected. To the degree that English cannot be used as a shared language between the research team and hospital staff, professional translators will be involved to support these activities.

### Study procedures

**Fig. 4:**
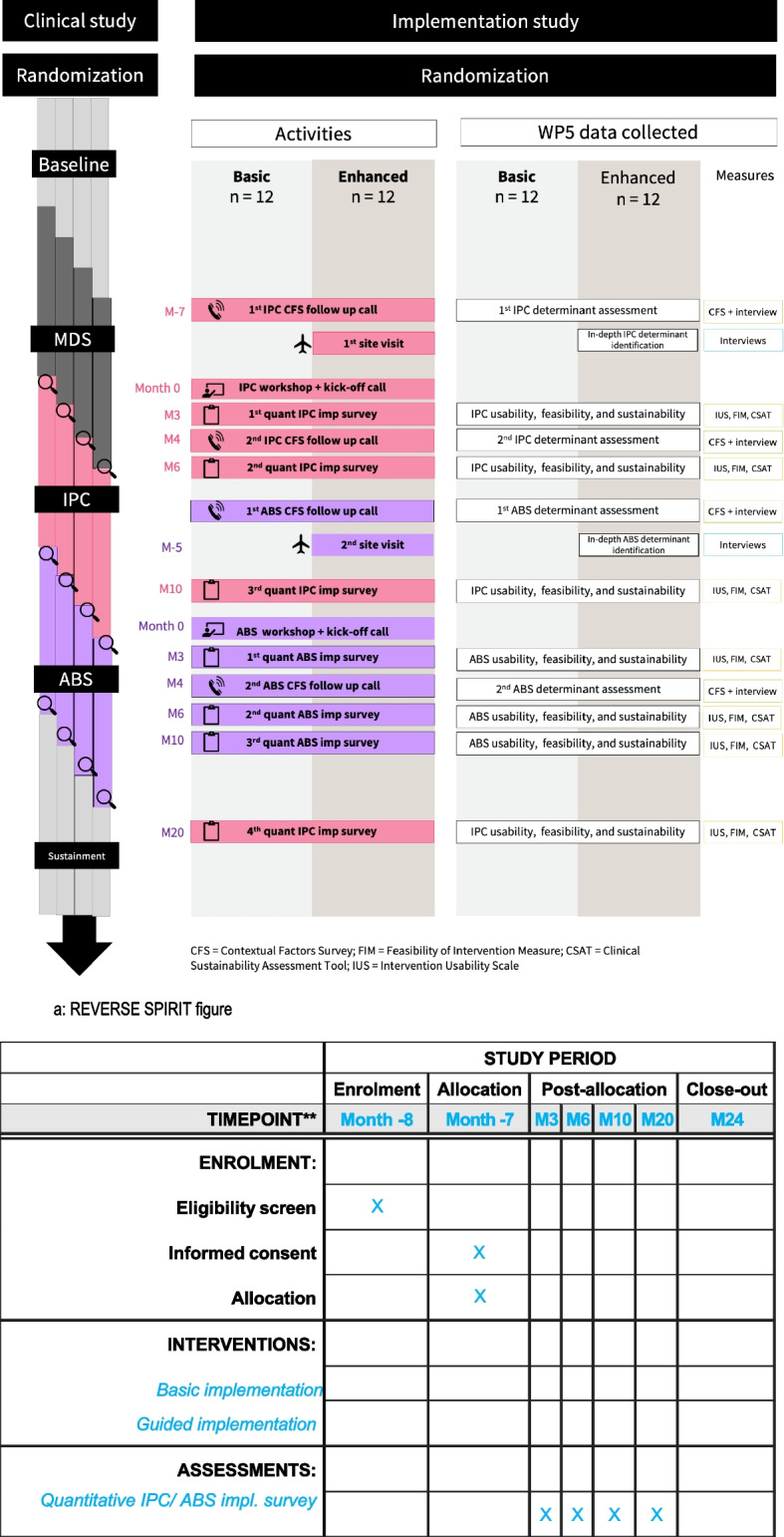
(**A**) The left part of this figure illustrates the sequential entering of cohorts into the trial. The middle column describes the data collection and implementation activities that the research team engages in with each cohort, and the right column lists the data collected as part of these activities. (**B**) REVERSE SPIRIT figure. A fully populated SPIRIT checklist for the REVERSE trial is provided in Supplementary Material 2 (Appendix B)

Based on the above, REVERSE implementation data collection activities will follow the chronology outlined in Fig. 4 a and b.

The left part of this figure illustrates the sequential entering of cohorts into the trial. The middle column describes the data collection and implementation activities that the research team engages in with each cohort, and the right column lists the data collected as part of these activities.

### Data analysis

#### Qualitative data analysis

To address our research question about IPC and ABS barriers and facilitators, qualitative data generated through individual interviews, CFS administration, follow-up interviews and implementation check-ins, and quarterly RITs will be analyzed using thematic analysis [63] based on open (i.e., inductive) as well as framework-informed (i.e., deductive) coding. To assess tailoring fidelity, RIT data representing information about locally identified implementation barriers and facilitators, as well as selected implementation strategies, together with any changes in implementation goal setting and scope, will be extracted and integrated into hospital-specific “tailoring profiles.” These will be used to assess the degree to which tailoring work occurred prospectively, was characterized by variation, and built on matching determinant-strategy pairs that are sufficiently operationalized. A traffic light system will be used to differentiate between low, medium, and high tailoring fidelity. Building on a multiple case study design, with each hospital representing a separate case, summaries of implementation determinants and theme identification will be conducted by hospital and country. Qualitative data will be coded independently by single researchers, who will be supported by a team of three researchers, who will jointly review, discuss, and refine identified codes and themes bi-weekly.

#### Quantitative data analysis

Multilevel modeling [64] with two analytic levels (Level 1: hospital staff, Level 2: hospital) will be conducted to investigate quantitative research questions forming the core of the REVERSE implementation evaluation. Multilevel models take into account the hierarchical data structure and the resulting non-independence of observations within groups and are suitable for dealing with unbalanced data, i.e., varying numbers of staff within each hospital [65]. To determine implementation outcomes, a null model will be fitted to each outcome variable to estimate the intercept, including a 95% confidence interval representing a point and interval estimate of the average outcome of interest. To determine tailoring effectiveness, outcomes will be predicted by group membership, investigating mean differences between basic vs. enhanced implementation while statistically controlling for the pretest scores and the effect of time to adjust for potential confounding of calendar time and intervention. All analyses will be conducted separately for months 3, 6, and 10 post-IPC and -ABS, and for month 20 post-IPC.

Restricted maximum likelihood estimation (REML) method with Kenward-Roger correction [66] will be used to estimate model parameters and standard errors. This method avoids small sample biases and has been shown to maintain the nominal Type-I error rates in data with Level 2 sample sizes below 25 [67]. All models will be estimated in R [68] using the *lme4* [69] and *lmerTest* [70] packages.

#### Data integration

Hospital-specific quantitative and qualitative data will be integrated, partly by using qualitative data to explain and put into perspective quantitative findings, including clinical and implementation outcomes, partly through conducting two to four retrospective, in-depth REVERSE explanatory case studies. Using guidance developed by Yin [71], these case studies will be aimed at developing explanations for hospital outcomes based on insights gained from exploring hospitals’ implementation work and experience over time, primarily represented in qualitative data. Cases will be selected near the end of data collection based on assessing their potential to contribute to a deeper understanding of REVERSE study outcomes. Hence, they may represent model, standard, critical, or, in another way, revelatory cases [72]. All data for a potential case will be brought together and examined by a single researcher, supported by a team of three researchers, who will jointly review and discuss case materials in light of overarching study findings and develop the case narrative.

## Discussion

REVERSE is an international, complex, and large-scale hybrid effectiveness-implementation trial designed to examine the effectiveness of guided tailoring in the implementation of practice bundles for strengthening infection prevention and control and antibiotic stewardship practices at 24 acute care hospitals in four European countries with a high prevalence of carbapenem-resistant organisms. Following recommendations for designing hybrid studies [31, 33], the REVERSE implementation evaluation is a hybrid type 2 study due to the need for a dual focus on evaluating intervention and implementation effectiveness. A thoroughly operationalized implementation strategy is critical to this type of design [31]. While this operationalization has occurred, considering the variety of barriers in different national and local settings, it remains unclear whether its essence—the ongoing monitoring and adjustment of implementation practice—will resonate and apply to any practice and any setting. With both the IPC and the ABS practice bundles allowing hospitals to select from a menu of target interventions that can be implemented across a multitude of specialties under very different local conditions, the potential variation in *what* hospitals implement *how* and *under which conditions* can be expected to be substantial. Furthermore, given that IPC and ABS practices will not be novelties for participating hospitals, their implementation of REVERSE practice bundles will commence from different baselines, adding further to this variation. While understanding usual conditions for practicing IPC and ABS is a focus point of REVERSE, these factors may nevertheless create uncertainties around the generalizability of study findings and, therefore, should be considered as part of their interpretation.

Attempts to enable tailoring have been at the center of other large-scale trials conducted in European healthcare settings, including, for example, the Tailored Implementation in Chronic Diseases (TICD) project [73, 74] and the ImpleMentAll project [46] focused on the tailored implementation of internet-based cognitive behavioral therapy. Among the key insights gained through these trials is the high degree of difficulty associated with practicing tailoring in complex settings with many competing priorities and preferences [73] and the potential importance of providing a minimum of support and facilitation [49]. REVERSE will build and add to this knowledge base by integrating facilitation into its overarching implementation approach. However, this also creates the potential challenge to disentangle tailoring from facilitation implementation strategies when interpreting study results. We will aim to address this challenge by keeping facilitation to what we perceive as a minimum; however, this facilitation may still explain part of the findings.

With implementation research being a relatively young scientific discipline, robust measurement instruments with strong psychometric properties remain scarce [75, 76]. This is reflected in the use of instruments for the REVERSE implementation evaluation, which builds on relatively new measures [54–56], all of which were developed in the USA. The international experience with using these measures is especially limited, as reflected in the fact that some need to be translated into different REVERSE languages. This implies that the sensitivity with which these instruments capture changes remains to be determined until further validation. This will be taken into account during data analyses.

The cultural diversity of the REVERSE project also implies that all data collection will occur under conditions of language barriers. While most instruments and tools used in the study are available in the four REVERSE languages, and translators will be involved in all site visit activities, it cannot be ruled out that connotations, nuances, and meaning get lost in translation processes, especially as part of interviews and other direct exchanges between researchers and study participants. To minimize these risks, quality standards for measure translation [57] and the conduct of cross-language research [77] will be applied to the REVERSE implementation evaluation, including thoroughly reporting translation decisions and translator use at the dissemination stage of the study [78].

As such, the REVERSE implementation trial will face several research challenges arising naturally from the rather heterogeneous conditions under which it will be conducted. By addressing these with nimble strategies and attention to detail, we believe that REVERSE is well-positioned to meaningfully contribute to the still scarce knowledge base on tailoring and thereby help strengthen current IPC and ABS practices in Europe.

## Trial status

The REVERSE trial runs from July 2021 to June 2026. Using purposeful sampling, acute care hospitals in the four REVERSE countries were recruited before the first baseline phase, which was by April 2022. The Zurich Cantonal Ethics Committee granted ethical approval for the REVERSE trial.

Implementation sites, in collaboration with country-specific National Focal Points (NFPs) teams, obtained local ethical approval from all involved hospitals, supported the translation of measurement instruments, and continue to facilitate local data collection.

With the commencement of REVERSE IPC implementation for the first cohort of hospitals in April 2023, each hospital began recruiting multiple implementation team members and ward staff to participate in the trial. This recruitment is open and will continue throughout the trial until the collection of implementation data seizes by the end of 2025. The first implementation evaluation results are expected to become available in 2026.

## Supplementary Information

Supplementary Material 1. Appendix A: Implementation strategy specification based on Proctor et al. 2013

Supplementary Material 2. Appendix B: REVERSE SPIRIT Checklist

## Data Availability

Translated versions of the measurement instruments *Feasibility Implementation Measure* (FIM), *Intervention Usability Scale* (IUS), and *Clinical Sustainability Assessment Tool* (CSAT) will be available upon request.

## Abbreviations

ABS: Antibiotic stewardship
AMR: Antimicrobial resistance
CFS: Contextual Factors Survey
CRAB: Carbapenem-resistant Acinetobacter baumannii
CRE: Carbapenem-resistant Enterobacterales
CRO: Carbapenem-resistant organism
CRPA: Carbapenem-resistant Pseudomonas aeruginosa
CSAT: Clinical Sustainability Assessment Tool
CST: Context-Specific Tailoring Model
EBP: Evidence-Based Practice
FIM: Feasibility of Intervention Measure
HAI: Healthcare-associated infections
IPC: Infection Prevention and Control
IUS: Intervention Usability Scale
MDRO: Multidrug-resistant organism
MDS: Microbiology and Diagnostic Stewardship
NFP: National Focal Point
SOP: Standard Operating Procedure

## Footnotes

1 https://www.reverseproject.eu/

2 https://www.isrctn.com/ISRCTN12956554

3 San Jose, CA: Zoom Video Communications Inc.
