# Supplementary material for: Examining tailoring as an implementation strategy for reducing healthcare-associated infections across European acute care hospitals (REVERSE): study protocol for a hybrid type 2 effectiveness-implementation trial
Source: Trials. 2025 Oct 16;26:418. doi: 10.1186/s13063-025-09132-x (PMC12532843; doi:10.1186/s13063-025-09132-x)
Supplement: Supplementary file 1 — Supplementary Material 1. Appendix A: Implementation strategy specification based on Proctor et al. 2013 [file 13063_2025_9132_MOESM1_ESM.docx]

**Appendix A**: Implementation strategy specification based on Proctor et al. 2013 [1]

| **REVERSE implementation strategy** | ***Basic cohort training*** | ***Curated collection of implementation resources*** | ***Forming a local implementation team*** | ***REVERSE kick-off calls*** | ***REVERSE implementation calls*** | ***Regular implementation reporting*** | ***Implementation check in*** |
| --- | --- | --- | --- | --- | --- | --- | --- |
| **Corresponding ERIC strategy** | Conduct educational meetings | Develop educational materials | Organize clinician implementation team meetings | Conduct educational meetings | Facilitation | Develop a formal implementation blueprint | Facilitation |
| **Condition included** | Basic  Enhanced | Basic  Enhanced | Basic  Enhanced | Basic  Enhanced | Enhanced | Basic (without feedback)  Enhanced (with feedback) | Basic  Enhanced |
| **Targets of the action – who?** | Clinicians centrally involved in a hospital’s work with IPC and/ or ABS | | | | | | |
| **Targets of the action – what?** | Knowledge about the practice bundles and current best implementation/ tailoring practice – skills in both areas. Intentions to implement practice bundles. | Knowledge about the practice bundles and current best implementation/ tailoring practice. | Intentions to implement practice bundles. Knowledge and skill in implementing practice bundles locally. Peer influences. | Knowledge about the practice bundles and current best implementation/ tailoring practice – skills in both areas. Intentions to tailor practice bundle implementation.  Intentions to implement practice bundles. Peer influences. | Implementation skill building – enhancing teams’ ability to tailor implementation, i.e., to reflect on, and plan for local implementation conditions and to solve implementation problems. Peer influences. | Implementation skill building – enhancing teams’ ability to tailor implementation, i.e., to reflect on, and plan for local implementation conditions and to solve implementation problems. Intentions to implement practice bundles. | Implementation skill building – enhancing teams’ ability to tailor implementation, i.e., to reflect on, and plan for local implementation conditions and to solve implementation problems. Intentions to implement practice bundles. |
| **Justification** | The strategy builds on core components of transformative learning theory [2,3] with the aim to sensitize adult learners to the importance of implementation informed thinking and acting in the context of infection prevention and antibiotic stewardship work. There is an emphasis on combining basic knowledge building (through basic training, educational resources and kick off calls) with opportunities for ongoing context specific critical reflection and discourse (through implementation teams and calls, regular reporting and feedback and implementation check ins). For the basic implementation condition the latter elements are used with reduced intensity. | | | | | | |
| **Actor(s)** | A team of eight-ten REVERSE researchers combining clinical (i.e., IPC or ABS) with implementation science expertise | | Central IPC and/ or ABS staff at each hospital | A team of two-four REVERSE researchers combining clinical (i.e., IPC or ABS) with implementation science expertise | | | Two members of the REVERSE implementation research team |
| **Action(s)** | Provide a mix of in-person didactic and interactive educational sessions to introduce the practice bundle, and principles of implementation best practice; to encourage initial hospital-based discussions about practice bundle implementation; and to answer questions as well as provide advice and encouragement | Share a collection of electronic materials about practices bundles and their implementation to support ongoing learning. Continually expand the collection with additional resources upon demand. | Consider who are central actors in facilitating, coordinating, progressing the work with IPC/ABS at the hospital. Bring these actors together on a regular basis to discuss actions and problem solving necessary to pursue collective goals set for both practice bundles. Define team mandate as well as roles and responsibilities for all team members. Secure team access to leadership support. | Gather three hospitals in the same implementation condition for an online meeting lasting 60-90 minutes to introduce the REVERSE Implementation Tool (RIT) to be used for ongoing tailoring and implementation monitoring. Discuss first implementation goals and ways to locally commence implementation work. Respond to any open questions. | Hold three online meetings focused on discussing three central elements in implementation (1: goal setting and determinant identification; 2: determinants and strategy selection; 3: strategy operationalization and implementation monitoring) based on attendees work with each of these areas. Provide advice, respond to questions, share tools and resources, provide encouragement | Quarterly submissions of updated versions of the REVERSE Implementation Tool (RIT) summarizing recent implementation activities and results.  As a standard, the implementation research team returns RIT with feedback, commentary, and advice within two-three weeks after submission. | One 60 minutes online meeting with the hospital’s implementation team with a focus on how practice bundle implementation is perceived, progresses, and can be strengthened. |
| **Temporality** | Hold in the month prior to commencing practice bundle implementation. | Share in the week post basic cohort training. | Within six weeks post basic cohort training. | Hold two-three weeks post basic cohort training. | Commences two-three weeks post kick off call. | First preliminary RIT to be submitted 6 weeks post kick off call. | Hold six months into practice bundle implementation. |
| **Dose** | Hold twice (prior to IPC, and ABS) for one cohort at a time, i.e., four IPC workshops and four ABS workshops in total. Each workshop lasts 1,5 days. | Constantly available to all implementation team members. | Forming of the team occurs twice, once for IPC, once for ABS. | Hold once (60-90 minutes call) for hospitals in the same implementation condition in each cohort, i.e., two calls per cohort. | Hold three weeks post kick-off call (call 1); six weeks post kick-off call (call 2); nine weeks post kick-off call (call 3); Each meeting involves those three hospitals in a cohort that are in the enhanced condition and lasts 60-90 minutes. Further calls can be requested on demand. | Quarterly submission per hospital. | Held once (60-90 minutes online meeting) per hospital |
| **Implementation outcomes affected** | Uptake of the practice bundle (adoption). Adherence to the protocol for the practice bundle (intervention fidelity) and to tailoring principles (implementation fidelity). | Adherence to the protocol for the practice bundle (intervention fidelity) and to tailoring principles (implementation fidelity). | Uptake of the practice bundle (adoption).  Capacity to maintain practice bundle implementation over time (sustainability). | Uptake of the practice bundle (adoption). Adherence to the protocol for the practice bundle (intervention fidelity) and to tailoring principles (implementation fidelity). | Uptake of the practice bundle (adoption). Adherence to the protocol for the practice bundle (intervention fidelity) and to tailoring principles (implementation fidelity). | Uptake of the practice bundle (adoption). Reach of practices across REVERSE wards (penetration). Adherence to the protocol for the practice bundle (intervention fidelity) and to tailoring principles (implementation fidelity). | Uptake of the practice bundle (adoption). Reach of SOPs across REVERSE wards (penetration). Maintenance of changes already implemented (sustainability). |

[1] Proctor EK, Powell BJ, McMillen JC. Implementation strategies: recommendations for specifying and reporting. Implementation Science 2013;8. <https://doi.org/10.1186/1748-5908-8-139>.

[2] Rojo J, Ramjan L, George A, Hunt L, Heaton L, Kaur A, et al. Applying Mezirow’s Transformative Learning Theory into nursing and health professional education programs: A scoping review. Teach Learn Nurs 2023;18:63–71. <https://doi.org/10.1016/j.teln.2022.09.013>.

[3] Mezirow J. An overview on transformative learning. Lifelong Learning, Routledge; 2006, p. 90–105.
